# Novel Kefir Exopolysaccharides (KEPS) Mitigate Lipopolysaccharide (LPS)-Induced Systemic Inflammation in Luciferase Transgenic Mice through Inhibition of the NF-κB Pathway

**DOI:** 10.3390/antiox12091724

**Published:** 2023-09-05

**Authors:** Chun-Huei Liao, Chih-Ching Yen, Hsiao-Ling Chen, Yu-Hsien Liu, Yu-Hsuan Chen, Ying-Wei Lan, Ke-Rong Chen, Wei Chen, Chuan-Mu Chen

**Affiliations:** 1 Department of Life Sciences, and Doctorial Program in Translational Medicine, National Chung Hsing University, Taichung 402, Taiwan; c9261011218@yahoo.com.tw (C.-H.L.); d5210@mail.cmuh.org.tw (C.-C.Y.); yuhsien000@yahoo.com.tw (Y.-H.L.); yhchen1218@smail.nchu.edu.tw (Y.-H.C.); coco.kinmen@gmail.com (K.-R.C.); 2 Division of Pulmonary Medicine, Department of Internal Medicine, China Medical University Hospital, Taichung 404, Taiwan; 3 College of Health Care, China Medical University, Taichung 404, Taiwan; 4 Department of Biomedical Science, Da-Yeh University, Changhua 515, Taiwan; hlchenbell@gmail.com; 5 Department of Internal Medicine, Jen-Ai Hospital, Dali Branch, Taichung 402, Taiwan; 6 Division of Pulmonary Biology, Cincinnati Children’s Hospital Medical Center, University of Cincinnati, Cincinnati, OH 45229, USA; bublelanwilliam@gmail.com; 7 Division of Pulmonary and Critical Care Medicine, Chia-Yi Christian Hospital, Chiayi 600, Taiwan; peteralfa2004@gmail.com; 8 The iEGG and Animal Biotechnology Center, National Chung Hsing University, Taichung 402, Taiwan; 9 Rong Hsing Research Center for Translational Medicine, National Chung Hsing University, Taichung 402, Taiwan

**Keywords:** kefir exopolysaccharides, LPS, NF-κB, MAPK, IL-6, transgenic mice, inflammation

## Abstract

A novel kefir exopolysaccharides (KEPS) derived from kefir grain fermentation were found to have a small molecular weight (12 kDa) compared to the traditionally high molecular weight (12,000 kDa) of kefiran (KE). KE has been shown to possess antioxidant, blood pressure-lowering, and immune-modulating effects. In this study, we characterized KEPS and KE and evaluated their anti-inflammatory properties in vitro using RAW264.7 macrophages. The main monosaccharide components were identified as glucose (98.1 ± 0.06%) in KEPS and galactose (45.36 ± 0.16%) and glucose (47.13 ± 0.06%) in KE, respectively. Both KEPS and KE significantly reduced IL-6 secretion in lipopolysaccharide (LPS)-stimulated macrophages. We further investigated their effects in LPS-induced systemic injury in male and female *NF-κB-luciferase^+/+^* transgenic mice. Mice received oral KEPS (100 mg/kg) or KE (100 mg/kg) for seven days, followed by LPS or saline injection. KEPS and KE inhibited NF-κB signaling, as indicated by reduced luciferase expression and phosphorylated NF-κB levels. LPS-induced systemic injury increased luciferase signals, especially in the kidney, spleen, pancreas, lung, and gut tissues of female mice compared to male mice. Additionally, it upregulated inflammatory mediators in these organs. However, KEPS and KE effectively suppressed the expression of inflammatory mediators, including p-MAPK and IL-6. These findings demonstrate that KEPS can alleviate LPS-induced systemic damage by inhibiting NF-κB/MAPK signaling, suggesting their potential as a treatment for inflammatory disorders.

## 1. Introduction

Kefir is a traditional fermented milk beverage made with kefir grains, which contain a complex mixture of lactic acid bacteria, acetic bacteria, and yeasts and are composed of kefir exopolysaccharides (KEPS), organic acids, lipid metabolites, protein complexes, and enriched peptides [1]. It is known for its extensive health benefits and diverse biological activities, including antimicrobial, immunomodulatory, antiallergenic, antitumoral, antidiabetic, and antioxidative effects [2,3]. Kefiran (KE) is typically prepared through the fermentation of *Lactobacillus kefiranofaciens* in a kefir-based medium. It has a molecular weight ranging from 100 to 100,000 kDa and possesses distinctive biochemical characteristics. KE is a water-soluble polysaccharide consisting of glucose, galactose, and mannose residues [4]. Its remarkable features include antimicrobial, antifungal, immunomodulatory, and antioxidant activities [5,6]. Due to these properties, KE holds great potential for a wide range of applications. It can be utilized in the food industry as a natural preservative, in pharmaceuticals for drug delivery systems, and in biomedical research for its potential immunotherapeutic and wound-healing properties [7,8]. KE’s multifaceted nature and beneficial attributes make it an intriguing candidate for further exploration in various scientific fields.

Systemic inflammation is a complex process involving dysregulated immune responses and the release of pro-inflammatory mediators, contributing to the pathogenesis of various diseases [9]. Activation of immune cells, such as macrophages and dendritic cells, triggers the production of cytokines, chemokines, and inflammatory mediators, resulting in the recruitment and activation of other immune cells. Dysregulation of the nuclear factor-kappa B (NF-κB) signaling pathway plays a critical role in driving systemic inflammation by promoting the expression of pro-inflammatory genes [10,11].

Systemic inflammation is associated with several diseases, including sepsis, rheumatoid arthritis, inflammatory bowel disease, and atherosclerosis. Sepsis is characterized by a dysregulated host response to infection, leading to widespread inflammation and organ dysfunction [12]. Rheumatoid arthritis is an autoimmune disease characterized by chronic inflammation and joint destruction [13]. Inflammatory bowel disease, including Crohn’s disease and ulcerative colitis, involves chronic inflammation of the gastrointestinal tract [14]. Atherosclerosis is a chronic inflammatory disease characterized by the accumulation of plaques in arterial walls [15]. Understanding the underlying mechanisms of systemic inflammation is crucial for developing targeted therapeutic interventions. Modulating the immune response, targeting pro-inflammatory mediators, and inhibiting the NF-κB signaling pathway are potential strategies for attenuating systemic inflammation and mitigating its associated diseases.

Natural-derived polysaccharides from plants, mushrooms, and seaweeds exhibit inhibitory effects on systemic inflammation through multiple mechanisms [16]. These polysaccharides interact with immune cells, such as macrophages and dendritic cells, leading to the downregulation of pro-inflammatory cytokines and the promotion of anti-inflammatory mediators. Additionally, they can modulate the activity of NF-κB, a key regulator of inflammation, by inhibiting its translocation and DNA binding activity. Furthermore, natural-derived polysaccharides exert antioxidant effects, attenuating oxidative stress associated with inflammation.

This study aimed to characterize and analyze a novel small molecular weight KEPS obtained from whole kefir grain fermentation, as well as a traditional large molecular weight KE derived from kefir-based single isolate *Lactobacillus kefiranofaciens.* We investigated the anti-inflammatory properties of KEPS and KE on bacterial lipopolysaccharide (LPS)-induced systemic inflammation in male and female *NF-κB-luciferase^+/+^* transgenic mice. Additionally, we assessed the impact of KEPS and KE on different organs and genders of transgenic mice while measuring proteins involved in the NF-κB and mitogen-activated protein kinase (MAPK) pathways to understand the underlying molecular mechanisms for preventing LPS-induced systemic inflammation and injury.

## 2. Materials and Methods

### 2.1. Obtaining a Novel KEPS Extract from Kefir Grain Fermentation

Kefir powder was purchased from Phermpep Co., Ltd. (Taichung, Taiwan) [17]. The kefir powder was subjected to hot water extraction by mixing it with four volumes of deionized water and heating it at 100 °C for 4 h [18]. Subsequently, the mixture was centrifuged at room temperature at 5000× *g* for 30 min. The resulting supernatant was further centrifuged and filtered using 20% trichloroacetic acid (1:1) at 4 °C and 5000× *g* for 30 min [19]. Then, the supernatant was mixed with three volumes of 95% ethanol at 4 °C for 12 h to precipitate insoluble polysaccharides. After centrifugation at 4 °C and 5000× *g* for 30 min, the obtained insoluble pellet, referred to as kefir exopolysaccharides (KEPS), was lyophilized and stored at −20 °C until further use.

### 2.2. Microorganism, Fermentation Conditions and Extraction of KE

The strain used for kefiran (KE) production in this study was *Lactobacillus kefiranofaciens* subsp. *kefiranofaciens* ATCC 43761 [20]. The bacterium was activated in Man-Rogosa-Sharpe (MRS) broth medium (LAB 094, Heywood, UK) and propagated by transferring it twice into fresh MRS medium after 48 h of growth. Fermentations were conducted in 300 mL bottles at 30 °C under anaerobic conditions. The resulting fermented broth by *L. kefiranofaciens* was cooled on ice, centrifuged, and filtered using 20% trichloroacetic acid (1:1) at 4 °C and 5000× *g* for 30 min [21]. The supernatant containing exopolysaccharide was combined with three volumes of 95% ethanol at 4 °C for 12 h to precipitate insoluble polysaccharides. The resulting mixture was then centrifuged at 4 °C and 5000× *g* for 30 min, resulting in the formation of an insoluble kefiran pellet. This pellet was subsequently lyophilized and stored at −20 °C until further use.

### 2.3. Molecular Weight Measurement of KEPS and KE

The molecular weight of KEPS and KE was determined using high-performance size-exclusion chromatography (HPSEC) [22]. KEPS and KE powders were dissolved in deionized water and filtered through a 0.2 μm filter after ultrasonic degassing. Chromatographic separation was performed using a Shodex OHpak SB-805 HQ column (300 × 8 mm) connected to a precolumn of the same type. The mobile phase, deionized water, was filtered and degassed. The analysis employed a refractive index (RI) detector (L-2490, Hitachi, Tokyo, Japan) and pump (L-2130, Hitachi) with a flow rate of 0.7 mL/min. A 20 μL aliquot of the sample solution was injected for HPSEC analysis. Pullulan standards (Shodex, Kanagawa, Japan) ranging from 6.2 kDa to 805 kDa were used to generate an eight-point standard curve (6.2–805 kDa versus retention time) for the determination of the molecular weights of KEPS and KE, performed in triplicate.

### 2.4. Monosaccharide Composition in KEPS and KE

For the analysis of monosaccharide composition in KEPS and KE, a complete digestion of KEPS or KE into simple sugars was performed. Firstly, 150 mg of KEPS or KE was mixed with 10 mL of 2 M trifluoroacetic acid (Sigma-Aldrich Co., St. Louis, MO, USA) and heated in a 100 °C water bath for 10 h. The resulting solution was evaporated under a vacuum and re-dissolved in 5 mL of deionized water. The monosaccharides were derivatized using *p*-aminobenzoic acid ethyl ester (ABEE), following a previously described method [23]. Briefly, aliquots of the mixture or standard solution (30 μL) were combined with 120 μL of the ABEE reagent solution (Seikagaku Co., Tokyo, Japan). The mixture was vortexed, heated at 80 °C for 1 h, and then cooled to room temperature. To extract the derivatives, 600 μL of distilled water and 600 μL of chloroform were added. After vigorous mixing and centrifugation, 5 μL of the upper aqueous layer was subjected to HPLC analysis. An HPLC system (Hitachi Co., Kyoto, Japan) with a gemini C 18 column (Phenomenex C18 250 mm × 4.6 mm, particle size 10.0 µm) and a UV detector (L-2400) was used. The mobile phase consisted of 0.04 M potassium borate buffer (pH 8.9, solvent A) and 100% acetonitrile (solvent B) with an isocratic elution in a 90:10 ratio. The column temperature was set at 50 °C, and analysis was performed at a flow rate of 0.8 mL/min and monitored at 308 nm for 1 h. Ten sugar standards (Sigma-Aldrich Co., St. Louis, MO, USA), including glucose, mannose, galactose, arabinose, ribose, xylose, fucose, rhamnose, glucuronic acid, and galacturonic acid were used for reference. The relationship between the peak areas and the quantities of ABEE-derivatized monosaccharides exhibited a linear correlation within the range of 1 to 1000 pmol.

### 2.5. Cell Culture and MTT Assay

The mouse macrophage cell line RAW264.7 was cultured in Dulbecco’s modified Eagle medium (DMEM) supplemented with 10% fetal bovine serum (FBS), 4 mM L-glutamine, 100 μg/mL streptomycin, and 100 U/mL penicillin (Life Technologies, Waltham, MA, USA) at 37 °C in a 5% CO_2_ humidified atmosphere. The cells were cultured in T75 flasks at a density of 1 × 10^6^ cells/well and passaged every two days. The cytotoxicity of the exopolysaccharides (KEPS and KE) was evaluated by treating RAW264.7 cells with various concentrations of the kefir-derived exopolysaccharides. Cell viability was assessed using the 3-(4,5-dimethylthiazol-2-diphenyl)-2,5-tetrazolium bromide (MTT; Merck, Darmstadt, Germany) colorimetric assay, as previously described [24]. Non-cytotoxic doses of the exopolysaccharides were selected for further anti-inflammatory assessments.

### 2.6. Anti-Inflammation Assessments of KEPS and KE In Vitro

To assess the anti-inflammatory effects of exopolysaccharides, an in vitro experimental model was employed using RAW264.7 cells [25]. The RAW264.7 cells (1 × 10^6^ cells/well) were co-cultured with LPS (Sigma-Aldrich Co., St. Louis, MO, USA) at a final concentration of 1 μg/mL, along with different concentrations (2–1000 μg/mL) of KEPS or KE samples. The plates were incubated at 37 °C in a 5% CO_2_ humidified incubator for 24 h. The cell culture supernatants were collected and stored at −80 °C for subsequent analysis of inflammatory cytokines [26,27]. The concentration of IL-6 in the cell culture media was determined using a commercially available quantitative enzyme-linked immunosorbent assay (ELISA) kit (Abcam, Cambridge, MA, USA), following the instructions.

### 2.7. Animals

Male and female *NF-κB-luciferase^+/+^* transgenic mice were housed under standard conditions, including a 12-h light/dark cycle and access to a standard laboratory diet and water ad libitum, as previously described [28]. At eight weeks of age, the mice were randomly divided into five groups (*n* = 6): (1) control group, receiving no treatment as a negative control; (2) LPS group, treated with 12.5 mg/kg LPS alone; (3) KEPS/LPS group, treated with 12.5 mg/kg LPS and 100 mg/kg kefir exopolysaccharides (KEPS); (4) KE/LPS group, treated with 12.5 mg/kg LPS and 100 mg/kg kefiran (KE); and (5) ASA/LPS group, treated with 12.5 mg/kg LPS and 12.5 mg/kg acetylsalicylic acid (ASA) as a positive control. LPS-induced systemic inflammation was established through intraperitoneal administration. The treatment groups received oral administration of exopolysaccharides for seven days, followed by intraperitoneal administration of LPS. The mouse dosage of KEPS or KE is 100 mg/kg/day, which is equivalent to a consumption of 665.88 mg per day for a 60-kg human adult. The positive control group received ASA orally for 1 h before LPS administration. NF-κB-induced luciferase expression was assessed through in vivo bioluminescence imaging, and tissues were collected for histological examination and protein extraction as previously described [11,29]. All animal experiments were conducted in accordance with guidelines and approved by the Institutional Animal Care and Utilization Committee of National Chung Hsing University, Taiwan (IACUC No. 107-037).

### 2.8. Bioluminescence Imaging

Imaging was conducted using the IVIS Imaging System 200 Series (Xenogen Corp., Alameda, CA, USA) at maximum sensitivity. *NF-κB-luciferase^+/+^* transgenic mice were intraperitoneally injected with luciferin (Promega, Los Altos, CA, USA) at a dose of 150 mg/kg in 200 μL volume and anesthetized with isoflurane [30]. After a 5-min interval, the mice were positioned supine in the chamber and imaged for 90 s using the IVIS Imaging System. Photon counts were quantified using Living Image^®^ software Version 2.50 (Xenogen Corp.), and the signal intensity was reported as photons/s/cm^2^.

### 2.9. Western Blot Analysis

Inflammatory protein expressions in various tissues were analyzed using Western blotting [31]. Tissues (pulmonary, spleen, and liver) were homogenized in RIPA buffer (EMD Millipore, Billerica, MA, USA) and centrifuged at 12,000 rpm for 30 min at 4 °C for protein extraction. Fifty micrograms of protein were separated by SDS-PAGE, transferred to a polyvinylidene difluoride (PVD) membrane, and incubated with primary antibodies. After washing, the membranes were probed with peroxidase-conjugated secondary antibodies and visualized using an ECL detection system (Millipore Corporation, Billerica, MA, USA).

### 2.10. Statistical Analysis

Data are presented as the means ± standard deviations (SD). Group differences were analyzed using *t*-tests or Duncan’s multiple-range test. Statistical analyses were performed using SPSS statistical software for Windows version 20.0 (SPSS Inc., Chicago, IL, USA) and one-way ANOVA with Duncan’s test. A significance level of *p* < 0.05 was used to determine statistical significance.

## 3. Results

### 3.1. Molecular Weight and Monosaccharide Composition of KEPS and KE

**Figure 1:**
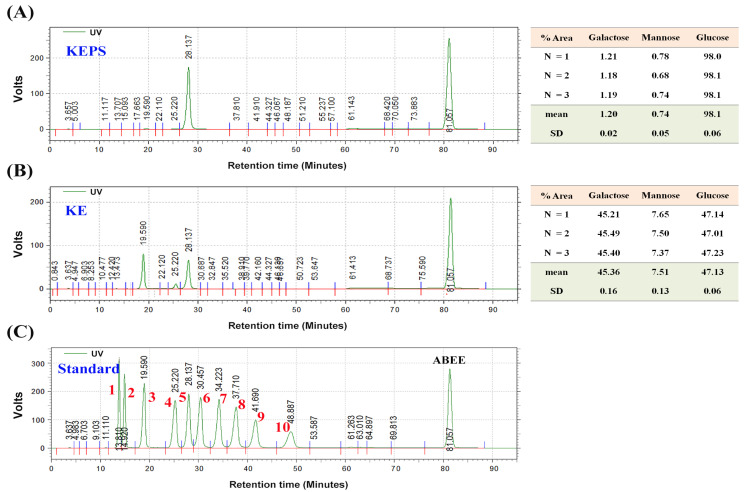
Monosaccharide composition of the kefir exopolysaccharides (KEPS) and kefiran (KE). A complete digestion of (**A**) KEPS and (**B**) KE into simple sugars was performed for the analysis of monosaccharide composition. (**C**) The retention times of ten sugar standards, including 1. glucuronic acid, 2. galacturonic acid, 3. galactose, 4. mannose, 5. glucose, 6. arabinose, 7. ribose, 8. xylose, 9. fucose, and 10. Rhamnose are provided as reference points for comparison. ABEE–*p*-aminobenzoic acid ethyl ester.

**Figure 2:**
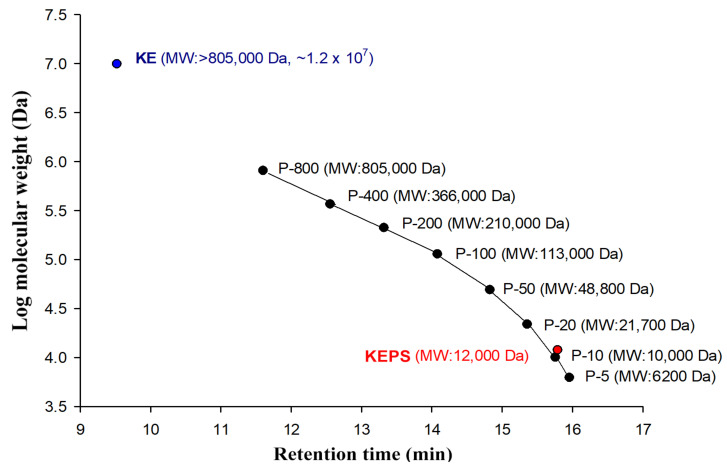
HPLC chromatograms for molecular weight measurement of kefir exopolysaccharides (KEPS) and kefiran (KE). The KEPS was prepared by alcohol precipitation hot water extracts from whole kefir grain fermentation. The KE was fermented by *L. kefiranofaciens* and prepared by alcohol precipitation. Pullulan standards (Shodex, Kanagawa, Japan) ranging from 6.2 kDa to 805 kDa were used to generate an eight-point standard curve.

The traditional natural fermented milk beverage, kefir, is widely recognized for its antimicrobial, antioxidative, and anti-inflammatory properties. However, the bioactive compounds in kefir’s hot water extracts remain poorly understood. In this study, we isolated a novel exopolysaccharide called KEPS from the hot water extracts of kefir. KEPS was obtained through alcohol precipitation from the fermentation of whole kefir grain, while a traditional kefiran (KE) was isolated through fermentation by a single lactobacterial strain, *L. kefiranofaciens*. The yield of KEPS and KE was 11.0 ± 0.4% (*w*/*w*) and 0.08 ± 0.01% (*w*/*v*), respectively. The neutral sugar compositions were primarily glucose (98.10 ± 0.06%) in KEPS, galactose (45.36 ± 0.16%), and glucose (47.13 ± 0.06%) in KE (Figure 1). HPLC analysis was conducted to further characterize KEPS and KE, revealing molecular weights of 12 kDa and 12,000 kDa, respectively (Figure 2).

### 3.2. Effect of the KEPS and KE Treatment on RAW264.7 Cells Viability

**Figure 3:**
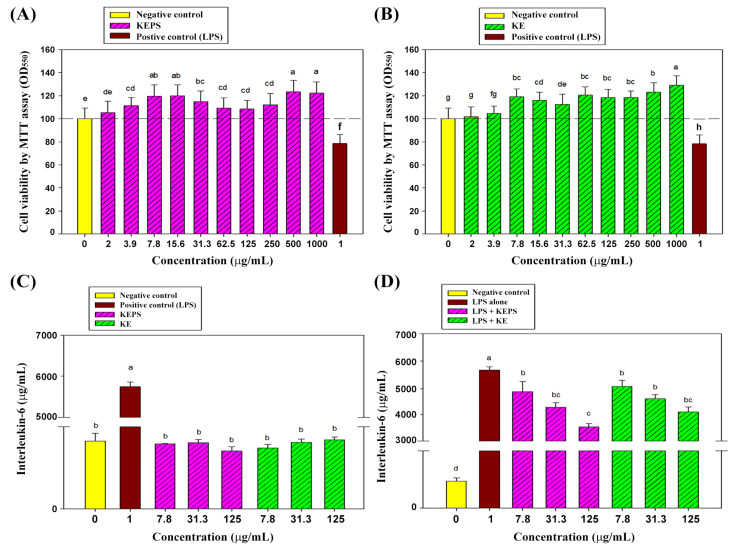
Effects of KEPS and KE treatments on cell viability and IL-6 cytokine secretion in RAW264.7 cells. RAW264.7 cell populations (1 × 10^6^ cells) were treated with different concentrations of KEPS (**A**) or KE (**B**) for 24 h, along with positive control of lipopolysaccharide (LPS) at a concentration of 1 μg/mL. Cell viability was assessed using the MTT assay. The effects of KEPS or KE treatment alone (**C**) or in combination with LPS (**D**) on IL-6 cytokine secretion by RAW264.7 cells were also examined. The data are presented as mean ± SD (*n* = 6). Bars with different letters indicate significant differences (*p* < 0.05) as determined by one-way ANOVA, followed by Duncan’s multiple-range tests. Cell viability (%) was calculated using the formula: [(*A*_sample_ − *A*_blank)_/(*A*_control_ − *A*_blank_)] × 100.

To assess the potential cytotoxic effects of KEPS and KE on RAW264.7 cells, various concentrations ranging from 0 to 1000 μg/mL were applied for 24 h. Cell viability was measured using the MTT assay, with LPS at 1 μg/mL serving as the internal control. The results demonstrated that both KEPS (Figure 3A) and KE (Figure 3B) treatments, except at the 2 μg/mL concentration, significantly accelerated the proliferation rates of RAW264.7 cells compared to the untreated control group (*p* < 0.05). In contrast, the LPS-treated group exhibited a 20% decrease in cell viability compared to the control group (*p* < 0.05). These findings suggest that KEPS and KE did not induce cytotoxicity at the tested concentrations. Moreover, both KEPS and KE displayed a stimulatory effect on cell proliferation at concentrations exceeding 2 μg/mL.

### 3.3. KEPS and KE Decreased the Pro-Inflammatory Cytokine Secretion in LPS-Induced RAW264.7 Cells

To investigate the anti-inflammatory potential of KEPS and KE on RAW264.7 cells in vitro, non-cytotoxic concentrations of 7.8, 31.3, and 125 μg/mL were used. After 24 h of treatment, the secretion of interleukin-6 (IL-6), a pro-inflammatory cytokine, was assessed. KEPS and KE treatments alone at the indicated concentrations did not significantly affect IL-6 secretion in the RAW264.7 cells (*p* > 0.05) (Figure 3C). However, when KEPS and KE were added to LPS-stimulated RAW264.7 cells for 24 h, a significant reduction in IL-6 secretion was observed (Figure 3D). KEPS treatment at 7.8, 31.3, and 125 μg/mL, as well as KE treatment at 31.3 and 125 μg/mL, resulted in a significant inhibition of IL-6 secretion by the LPS-stimulated RAW264.7 cells (*p* < 0.05) (Figure 3D). These findings indicate that KEPS and KE possess strong anti-inflammatory potential by decreasing IL-6 secretion in LPS-stimulated RAW264.7 macrophage cells.

### 3.4. KEPS and KE Inhibited the LPS-Induced NF-κB Activation in NF-κB-luciferase^+/+^ Transgenic Mice

**Figure 4:**
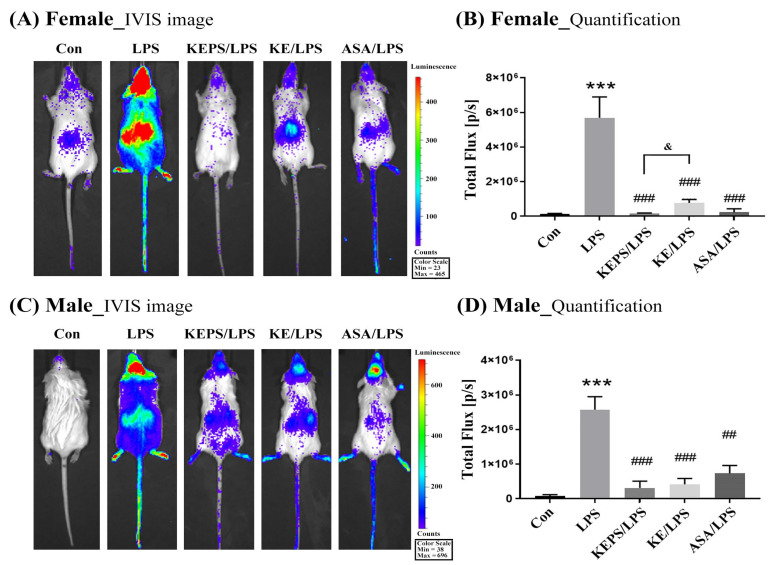
Effects of KEPS and KE on the bioluminescence signaling of LPS-induced inflammation in *NF-κB-luciferase^+/+^* transgenic mice. Control (Con) group received no treatment, serving as the negative control; LPS group was treated with 12.5 mg/kg LPS alone; KEPS/LPS group received oral administration of 100 mg/kg KEPS for 7 days, followed by treatment with 12.5 mg/kg LPS; KE/LPS group received oral administration of 100 mg/kg KE for 7 days followed by treatment with 12.5 mg/kg LPS; and ASA/LPS group was treated with 12.5 mg/kg LPS and 12.5 mg/kg acetylsalicylic acid (ASA) as the positive control. (**A**) The IVIS images of female *NF-κB-luciferase^+/+^* transgenic mice, and (**B**) the quantification data of bioluminescence signals in female mice. KEPS, KE, and ASA significantly reduced the LPS-induced bioluminescence signals in female mice compared to the LPS alone group. (**C**) The IVIS images of male *NF-κB-luciferase^+/+^* transgenic mice, and (**D**) the quantification data of bioluminescence signals in male mice. The data are expressed as the mean ± SD (*n* = 3). *** *p* < 0.001 compared to the control group. ^##^ *p* < 0.01, ^###^ *p* < 0.001 compared to the LPS group. ^&^ *p* < 0.05 compared to the KE/LPS group.

*NF-κB-luciferase^+/+^* transgenic mice express luciferase under the control of the NF-κB response element in the promoter, allowing for the assessment of NF-κB activity. To investigate the impact of LPS-induced systemic inflammation, transgenic mice were treated with 12.5 mg/kg LPS via intraperitoneal administration for 24 h. Luciferase signals were then quantified in bioluminescence images of the entire body using the in vivo imaging system (IVIS) in female mice (Figure 4A,B) and in male mice (Figure 4C,D). To explore the anti-inflammatory effect of kefir exopolysaccharides, the treatment groups underwent oral administration of exopolysaccharides (KEPS or KE) for a period of 7 days, which was followed by intraperitoneal administration of LPS. Results showed that the luminescent signals in female mice were significantly 2-fold higher (5.72 ± 1.31 × 10^6^ p/s) than in male mice (2.51 ± 0.45 × 10^6^ p/s) following LPS stimulation. However, the exopolysaccharide-treated groups (KEPS/LPS and KE/LPS) exhibited significantly lower luciferase signals compared to the LPS alone group (*p* < 0.001; Figure 4B,D). Notably, KEPS treatment was more effective than KE treatment in reducing luciferase signals, specifically in female mice (*p* < 0.05; Figure 4B).

**Figure 5:**
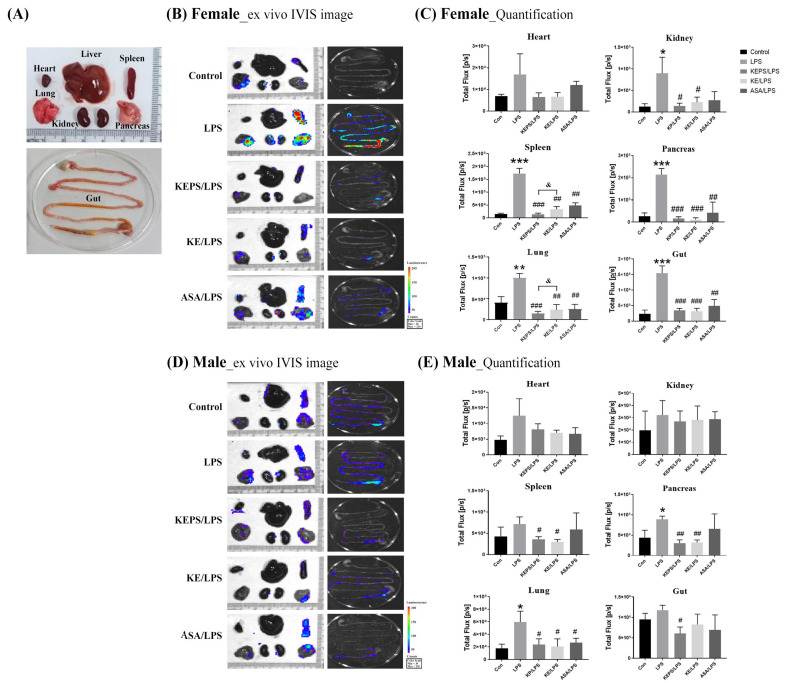
Effects of KEPS and KE on the ex vivo bioluminescence signaling of LPS-induced inflammation in various organs of *NF-κB-luciferase^+/+^* transgenic mice. (**A**) The arrangement of mouse organs in a bright field for ex vivo IVIS imaging. (**B**) The representative IVIS images of different organs in female *NF-κB-luciferase^+/+^* transgenic mice. (**C**) The quantification data of bioluminescence signals in various organs of female mice, including the heart, kidney, spleen, pancreas, lung, and gut. (**D**) The representative IVIS images of different organs in male *NF-κB-luciferase^+/+^* transgenic mice. (**E**) The quantification data of bioluminescence signals in various organs of male mice. The data are presented as mean ± SD. (*n* = 3). Statistical significance is indicated as * *p* < 0.05, ** *p* < 0.01, *** *p* < 0.001 compared to the control group, ^#^ *p* < 0.05, ^##^ *p* < 0.01, ^###^ *p* < 0.001 compared to the LPS alone group, and ^&^ *p* < 0.05 compared to the KE/LPS group.

To determine the specific organs affected by LPS induction, bioluminescence imaging was performed on ex vivo internal organs of *NF-κB-luciferase^+/+^* transgenic mice (Figure 5A). In female mice, LPS administration significantly increased the luminescent signal in various internal organs, including the spleen, lung, kidney, pancreas, and gut tissue, compared to the untreated control (Figure 5B). However, the KEPS/LPS, KE/LPS, and ASA/LPS groups exhibited significantly lower luciferase signals compared to the LPS alone group (Figure 5C). In male mice, LPS only significantly increased the luminescent signal in the lung and pancreas (Figure 5D), but the KEPS/LPS and/or KE/LPS groups showed significantly lower luciferase signals in the spleen, lung, and pancreas compared to the LPS alone group (Figure 5E). Therefore, treatment with 100 mg/kg body weight of exopolysaccharides, either KEPS or KE, significantly protected *NF-κB-luciferase^+/+^* transgenic mice against systemic inflammation induced by LPS exposure.

### 3.5. KEPS and KE Decreased the Inflammatory Mediator p-NF-κB Activation in LPS-Induced NF-κB-luciferase^+/+^ Transgenic Mice

**Figure 6:**
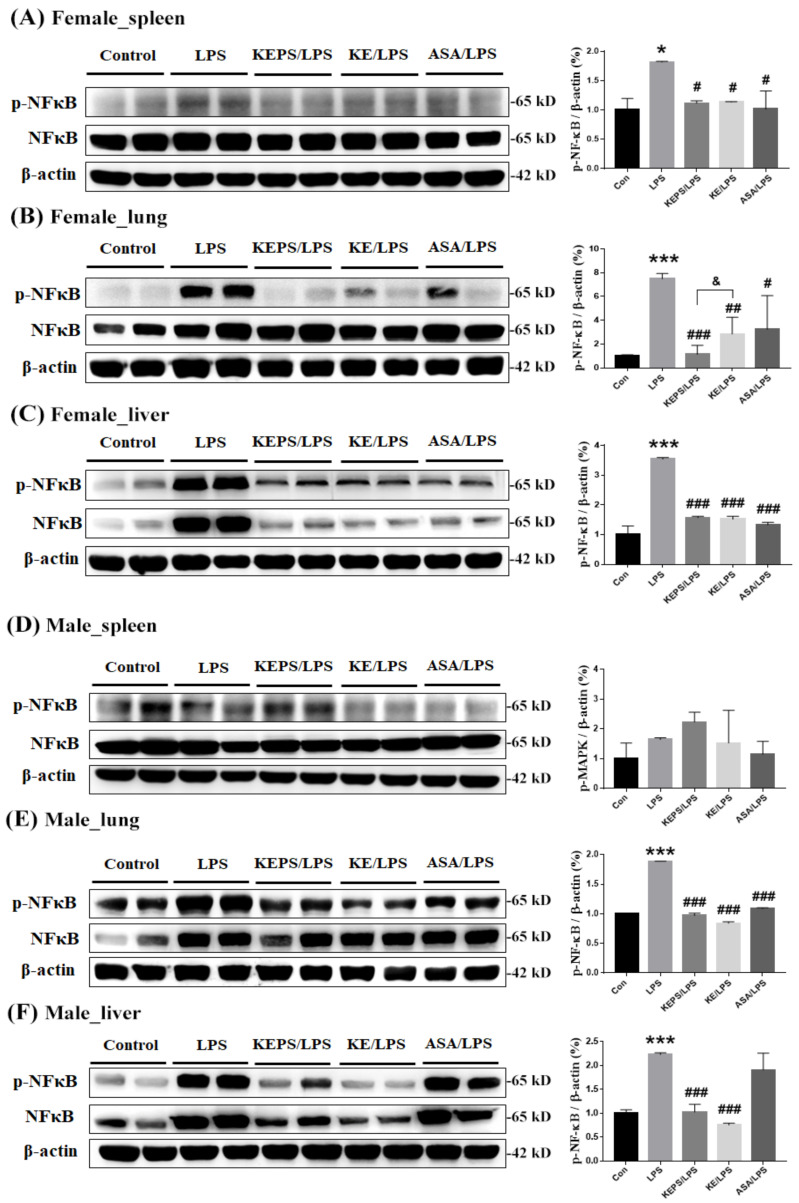
Effects of KEPS and KE on alleviating LPS-induced inflammation in various organs of *NF-κB-luciferase^+/+^* transgenic mice by inhibiting phosphorylated NF-κB (p-NF-κB) expression. Representative images of western blots analysis of total NF-κB and phosphorated NF-κB protein expression in (**A**) spleen, (**B**) lung, and (**C**) liver tissues in female mice and (**D**) spleen, (**E**) lung, and (**F**) liver tissues in male mice. The data are presented as mean ± SD (*n* = 3). Statistical significance is indicated as * *p* < 0.05, *** *p* < 0.001 compared to the control group, ^#^ *p* < 0.05, ^##^ *p* < 0.01, ^###^ *p* < 0.001 compared to the LPS alone group, and ^&^ *p* < 0.05 compared to the KE/LPS group.

To assess LPS-induced systemic inflammation in female and male transgenic mice, total NF-κB and phosphorylated p-NF-κB levels were measured in spleen, lung, and liver tissues (Figure 6). In female mice, LPS significantly increased p-NF-κB expression in the spleen (*p* < 0.05), lung (*p* < 0.001), and liver (*p* < 0.001) compared to the control group (Figure 6A, 6B, and 6C, respectively). Treatment with KEPS, KE, or ASA significantly reduced p-NF-κB expression in all examined organs. In male mice, LPS significantly increased p-NF-κB expression in the lung (*p* < 0.001; Figure 6E) and liver (*p* < 0.001; Figure 6F) compared to the control group. KEPS, KE, and ASA reduced p-NF-κB expression in the lungs of male mice, while KEPS and KE reduced total NF-κB and p-NF-κB expressions in the liver of male mice, but not in the ASA-treated group (Figure 6F).

### 3.6. KEPS and KE Decreased the Inflammatory Mediator p-MAPK Expression in LPS-Induced NF-κB-luciferase^+/+^ Transgenic Mice

**Figure 7:**
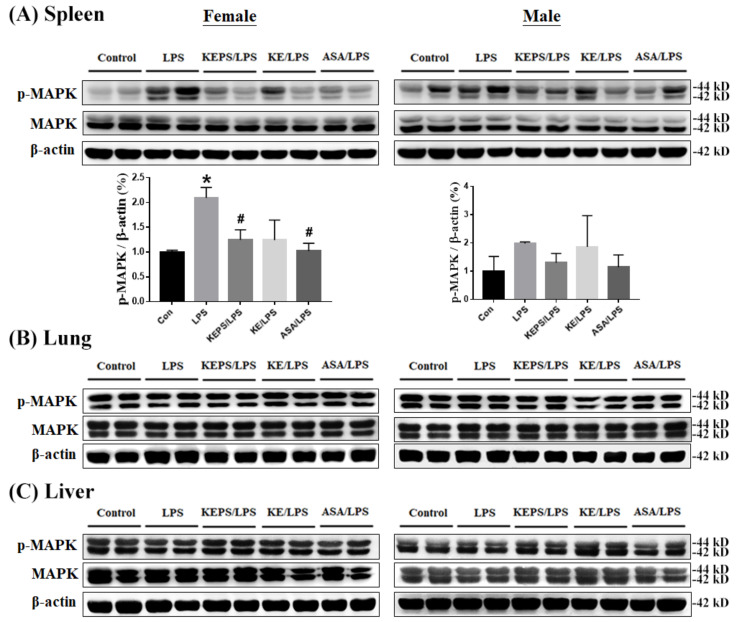
Effects of KEPS and KE on alleviating LPS-induced inflammation in various organs of *NF-κB-luciferase^+/+^* transgenic mice by inhibiting phosphorylated MAPK (p-MAPK) expression. Representative images of western blots analysis of total MAPK and p-MAPK protein expression in (**A**) spleen, (**B**) lung, and (**C**) liver tissues in female and male mice, respectively. β-actin was used as an internal control. The data are presented as mean ± SD (*n* = 3). Quantification data is shown for spleen samples. Statistical significance is indicated as * *p* < 0.05 compared to the control group and ^#^ *p* < 0.05 compared to the LPS alone group.

Intriguingly, the expression of p-MAPK protein was significantly increased in the spleen of female mice in the LPS alone group compared to the control group (Figure 7A) but not in the lung (Figure 7B) and liver (Figure 7C) organs. KEPS and ASA significantly reduced p-NF-κB expression in the spleen of female mice, while KE treatment did not show a significant effect (Figure 7A). In male mice, both total MAPK and phosphorylated p-MAPK levels were not significantly affected by LPS induction in the spleen (Figure 7A), lung (Figure 7B), and liver (Figure 7C). These findings suggest that female mice exhibit greater sensitivity to p-MAPK expression in the spleen after LPS induction compared to male mice.

### 3.7. KEPS and KE Decreased the Serum Pro-Inflammatory Cytokine IL-6 Content in LPS-Induced NF-κB-luciferase^+/+^ Transgenic Mice

**Figure 8:**
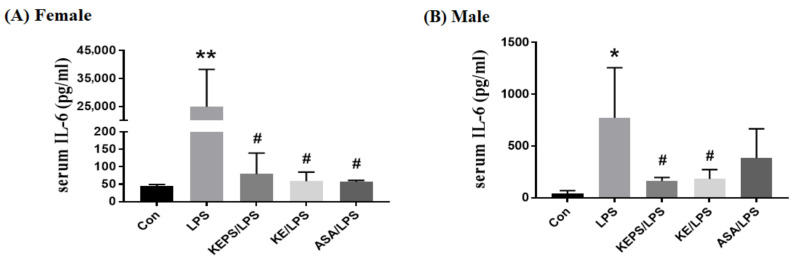
Effects of KEPS and KE on the alleviation of LPS-induced serum IL-6 inflammatory cytokine in *NF-κB-luciferase^+/+^* transgenic mice. ELISA assay was performed to measure the suppression of LPS-induced IL-6 inflammatory cytokine by KEPS, KE, and ASA in female (**A**) and male (**B**) mice. The data are presented as mean ± SD (*n* = 3). Statistical significance is indicated as * *p* < 0.05, ** *p* < 0.01 compared to the control group, and ^#^ *p* < 0.05 compared to the LPS alone group.

Serum levels of the pro-inflammatory cytokine IL-6 were significantly elevated in LPS-induced *NF-κB-luciferase^+/+^* transgenic mice (Figure 8). Specifically, the serum IL-6 concentration in female mice (25,106 ± 11,075 pg/mL; Figure 8A) was 30-fold higher than that in male mice (785 ± 505 pg/mL; Figure 8B) in the LPS alone group. Treatment with KEPS, KE, and ASA significantly reduced IL-6 levels in female mice (*p* < 0.001; Figure 8A), while only KEPS and KE treatments effectively decreased IL-6 levels in male mice (*p* < 0.05; Figure 8B).

## 4. Discussion

In this study, we made three key observations demonstrating the anti-inflammatory effects of KEPS and KE in alleviating LPS-induced systemic inflammation in *NF-κB-luciferase^+/+^* transgenic mice by inhibiting the NF-κB/MAPK pathway. Firstly, KEPS and KE exhibited molecular weights of approximately 12 and 12,000 kDa, respectively, with KEPS primarily composed of glucose and KE composed of galactose and glucose. Secondly, in vitro experiments showed that KEPS and KE significantly reduced the secretion of pro-inflammatory cytokine IL-6 in LPS-stimulated RAW264.7 macrophages, indicating their anti-inflammatory properties. Lastly, exposure to LPS for 24 h intraperitoneally successfully induced systemic inflammation in transgenic mice, particularly in females. This exposure led to the upregulation and phosphorylation of inflammatory mediators in the spleen, lung, and liver tissues through activation of the NF-κB/MAPK pathway. However, oral administration of both KEPS and KE significantly suppressed this response.

LPS is a bacterial cell wall phospholipid that triggers inflammation and shares similarities with septic shock. Systemic inflammation caused by LPS exhibits variations in acute organ damage and gender differences, highlighting the need for further investigation into the underlying mechanisms. Different organs, such as the liver, lung, spleen, and kidney, display distinct patterns of LPS-induced inflammation [32,33]. In the liver, acute inflammation is characterized by increased production of pro-inflammatory cytokines like tumor necrosis factor-alpha (TNF-α) and IL-6, along with activation of inflammatory signaling pathways [34,35]. Liver injury also involves apoptosis, leading to specific changes such as chromosomal DNA fragmentation and caspase activation [36]. In the lungs, LPS recognition by Toll-like receptor 4 (TLR4) on bronchial epithelial cells and lung macrophages contributes to acute lung injury (ALI) and triggers innate immune responses, the release of inflammatory mediators, and activation of the NF-κB pathway [37]. LPS-induced lung injury may further result in respiratory dysfunction and impaired gas exchange [38].

In this study, we observed a higher susceptibility to LPS-induced inflammation in adult female mice compared to males through analysis of whole-body bioluminescence images (Figure 4), expression of inflammatory mediators in organs (Figure 6 and Figure 7), and serum levels of the pro-inflammatory cytokine IL-6 (Figure 8). Sexual dimorphism has been reported in the response to LPS-induced inflammation, where females exhibited a more pronounced inflammatory response compared to males [39]. This suggests a potential role of sex hormones in modulating immune reactions, where estrogen may impact the production of pro-inflammatory cytokines and chemokines, promote the recruitment and activation of immune cells, and regulate the expression of immune receptors and signaling molecules. In contrast, testosterone, the primary male sex hormone, may have immunosuppressive effects by attenuating immune responses and reducing the production of pro-inflammatory mediators [40]. Furthermore, sex differences in the severity and mortality of infectious and inflammatory diseases in premature animals and children are primarily determined by the karyotype rather than sex hormones. Notably, certain immune response proteins, including those in the NF-kB signaling pathway, are encoded on the X chromosome [41,42].

Understanding the complex mechanisms underlying LPS-induced systemic inflammation in various organs and between sexes has important clinical implications. This knowledge may assist in the development of targeted therapeutic strategies for managing conditions like sepsis, irritable bowel syndrome (IBS), osteoarthritis (OA), and retinal neurodegenerative diseases. By targeting specific pathways involved in systemic inflammation, these strategies have the potential to improve patient outcomes and alleviate the burden of diseases associated with systemic inflammation [43,44]. IL-6 signaling occurs via two mechanisms: binding to membrane-bound IL-6 receptor (mbIL6R) and interaction with soluble IL-6 receptor (sIL6R) [34]. IL-6 binding to mbIL6R recruits membrane-bound glycoprotein 130 (gp130), initiating downstream signaling through Janus kinases/signal transducer and activator of transcription (STAT) kinases, phosphoinositide 3-kinase (PI3K), and MAPKs such as p38 [45]. sIL6R is generated through mRNA splicing or proteolysis by disintegrin and metalloproteinase (ADAM) proteases [34]. IL-6, along with C-reactive protein (CRP), is commonly used as a serum biomarker to monitor inflammation in patients with cancer, infection, or autoimmune diseases due to its crucial role in activating and sustaining the inflammatory response [46].

This study identified organ-specific variations in LPS-induced systemic inflammation. Specifically, the bioluminescence signals of the gut organ in female mice were significantly higher compared to other organs and even male mice (Figure 5). However, due to autofluorescence background from dietary food, further examination of their inflammatory mediator expression was not conducted. Additionally, the bioluminescence signals of the heart and kidney organs in both gender and male mice, respectively, did not display significant alterations among different groups (Figure 5). Consequently, we focused on the study of three organs, including the spleen, lung, and liver. LPS-induced systemic inflammation in the spleen is important due to its role as a key immune organ involved in the response to bacterial infections. The spleen houses immune cells and plays a crucial role in pathogen clearance and immune activation [47]. LPS-induced lung injury involves the regulation of inflammatory mediators, including IL-6, NF-κB, and MAPK signaling pathways. LPS triggers the activation of NF-κB and MAPK, leading to the production of pro-inflammatory cytokines, such as tumor necrosis factor-alpha (TNF-α) and IL-6, which contribute to lung inflammation and injury [48]. The liver, on the other hand, plays a crucial role in detoxifying circulating LPS and clearing inflammatory mediators. However, excessive LPS exposure can overwhelm the liver’s capacity, resulting in liver inflammation and dysfunction [49]. Additionally, LPS-induced systemic inflammation can affect the cardiovascular system, leading to endothelial dysfunction and cardiovascular complications.

Exopolysaccharides (EPS) derived from different sources, such as marine organisms, fungi, and probiotic fermentation products, have shown promising effects in mitigating LPS-induced systemic inflammation. These EPS compounds possess distinct molecular weights, ranging from low (<10 kDa) to high (>100,000 kDa), which contribute to their functional properties [50]. Studies have revealed that EPS can modulate the production of inflammatory mediators, including IL-6, which plays a crucial role in the inflammatory response. EPS treatment has been shown to inhibit the expression and release of IL-6, thereby attenuating the systemic inflammatory cascade triggered by LPS [51,52]. Furthermore, EPS exerts its anti-inflammatory effects by targeting key signaling pathways involved in inflammation, such as NF-κB and MAPKs. EPS treatment has been found to inhibit NF-κB activation and suppress the phosphorylation of MAPKs, including p38, ERK, and JNK. These pathways are known to regulate the expression of pro-inflammatory cytokines and contribute to the development and progression of systemic inflammation [53].

In addition, previous investigations have showcased the remarkable antioxidant potential of EPS derived from *Bacillus* sp. in Thai milk kefir. This EPS exhibited significant antioxidant activities, as evidenced by a 57.5% free radical scavenging effect in the 2,2′-diphenyl-1-picrylhydrazyl (DPPH) radical scavenging assay, a value of 37.2 μM Fe(II)/mg EPS in the ferric reducing/antioxidant power (FRAP) assay, and a Trolox equivalent antioxidant capacity (TEAC) value of 34.9 μM [54]. Notably, the EPS-based kefiran biopolymer demonstrated superior reducing power and superoxide radical scavenging when compared to hyaluronic acid (HA) [5]. Moreover, fermented milk containing EPS from *L. plantarum* YW11 exhibited favorable antioxidant and gut microbiota regulatory effects [55]. These antioxidative properties align with numerous advantageous functions for human well-being, including the strengthening of the immune system [56]. These studies highlight the prospective applications of EPS in the development of antioxidant-rich functional foods, regenerative medicine, and whitening cosmetics.

In this study, we observed that the lower molecular weight of KEPS exhibited slightly greater efficacy in inhibiting LPS-induced systemic inflammation compared to KE in RAW264.7 macrophages (Figure 3) and various organs (Figure 6). It is known that high molecular weight EPS often possess stronger immunomodulatory abilities, as they can activate immune cells and enhance their anti-inflammatory response. On the other hand, low molecular weight EPS typically exhibits higher bioavailability and better cellular penetration [57]. Based on these characteristics, the novel isolated KEPS holds significant clinical promise. It can be applied in the treatment of chronic inflammatory diseases such as OA, rheumatoid arthritis (RA), and inflammatory bowel disease (IBD).

## 5. Conclusions

In summary, our study identified a novel small molecular weight KEPS derived from whole kefir grain fermentation. Both KEPS and KE demonstrated significant reductions in IL-6 secretion in LPS-stimulated macrophages in vitro. Using the *NF-κB-luciferase^+/+^* transgenic mouse model, we observed heightened luciferase signals in the spleen, lung, liver, and gut tissues of female mice compared to males, indicating LPS-induced systemic injury. However, oral administration of KEPS or KE for seven days effectively inhibited NF-κB signaling, as evidenced by decreased luciferase expression, serum IL-6 levels, and phosphorylated NF-κB and MAPK levels. These findings highlight the therapeutic potential of KEPS and/or KE in treating inflammatory disorders by mitigating LPS-induced systemic damage through the inhibition of NF-κB/MAPK signaling.

## 6. Patents

The research data presented in this study have been filed for a patent application with the Intellectual Property Office in Taiwan. The patent application number is 110120302.

## Data Availability

The data presented in this study are available in the article.
